# An Evil Consequence of Pince-Nez

**Published:** 1902-03-29

**Authors:** 


### AN EVIL CONSEQUENCE OF PINCE-NEZ.

Mr. Robert D. Joyce,[1] ophthalmic surgeon to the Richmond Hospital, Dublin, calls attention to a very curious point in regard to the use of the pince-nez, namely, that in some cases it sets up a flow of tears in consequence of its pressure dragging on the tissues at each side of the nose to such an extent as to pull the lower lid out of position, and prevent it lying properly in contact with the bulbar conjunctiva. In the cases observed, the displacement of the lid, with of course the inferior punctum, was small in amount, only about 0·2 or 0·3 mm. This might easily escape detection, and all the more so, as it has to be looked for while the glasses are on the patient's face. But even this small amount of eversion is amply sufficient to cause epiphora. This, he says, is a very important matter, for such an eversion of the lid, though present at first only when the pince-nez is actually on the face, will become permanent in time, and set going all the troubles associated with ectropion. In a large proportion of cases pince-nez will sit well on the face, but in a certain number they are unsuitable for the above reason, even though they may be a "good fit" so far as the optician is concerned. In some cases it is not possible to fit pince-nez without causing this kind of ectropion, and in such, of course, spectacles must be used.

[1] Brit. Med. Journ., March 22.

